# Durable Local Control Following Concurrent Hypofractionated Chemoradiation for a Massive Inflammatory Breast Cancer Chest Wall Recurrence

**DOI:** 10.7759/cureus.1379

**Published:** 2017-06-21

**Authors:** Brandon A Dyer, Ky Nam B Nguyen, Rakendu P Shukla, Tianhong Li, Megan E Daly

**Affiliations:** 1 Radiation Oncology, University of California Davis Comprehensive Cancer Center; 2 Hematology and Oncology, University of California Davis Comprehensive Cancer Center

**Keywords:** inflammatory breast cancer, local control, concurrent chemoradiation, hypofractionation, hypofractionated radiation, radiobiology, breast cancer recurrence, radiosensitization

## Abstract

Breast cancer is the leading new cancer diagnosis in women in the United States and is the second most lethal cancer in this patient population after lung cancer. Chest wall recurrence after mastectomy poses unique clinical challenges, as such tumors are often not amenable to surgical resection and durable local control with radiation or systemic therapy is challenging. When uncontrolled, chest wall recurrence can lead to severe pain and other morbidity. Herein, we describe a patient with inflammatory breast cancer with a massive, rapidly growing chest wall recurrence treated with a regimen of hypofractionated concurrent chemoradiation resulting in a complete chest wall response with durable local control.

## Introduction

Breast cancer is the leading new cancer diagnosis in women in the United States (US) comprising 29% of all female cancer diagnoses and is the second most lethal cancer in this patient population after lung cancer [1-2]. Despite improved screening and detection modalities, tumor molecular and genetic profiling, and systemic therapeutic options, the survival rates for locally advanced disease is 30–50% at 10 years and 30–40% at 20 years [3-5]. Trimodality therapy is the mainstay of treatment for advanced invasive disease not eligible for breast conservation [6]. Recurrent disease after mastectomy poses unique therapeutic challenges as such recurrences often cannot be surgically resected with acceptable morbidity. Herein, we describe a patient with inflammatory breast cancer treated initially with neoadjuvant chemotherapy and mastectomy with a rapidly growing, massive chest wall tumor recurrence treated with concurrent chemoradiation (CRT) with durable local control.

## Case presentation

A 63-year-old woman presented to our institution for management of locally recurrent breast cancer. She initially presented to an outside hospital with a palpable left breast mass eight months prior. Mammography at diagnosis showed a 4.7 cm posterocentral mass and ultrasound showed complex, cystic echotexture. Core needle biopsy was performed revealing poorly differentiated infiltrating ductal carcinoma (IDC) with lymphovascular invasion (LVI). Immunohistochemical staining showed the tumor to be estrogen receptor (ER) negative, progesterone receptor (PR) negative, receptor tyrosine-protein kinase erbB-2 (HER2) negative with a high proliferative index, Ki-67 of 33%. The clinical findings and histopathology were consistent with inflammatory breast cancer. Due to limited access to medical care, she had a long treatment delay and ultimately underwent staging computed tomography (CT) three months later, which revealed significant tumor progression with an 8 cm craniocaudal (CC) by 10 cm transverse (TRANS) by 7 cm anteroposterior (AP) mass involving the medial left breast with chest wall and skin involvement, bilateral moderate-sized pleural effusions, left greater than right, and confluent left axillary adenopathy beginning in low axillary level I through level III and measuring up to 3 cm in maximum dimension without evidence of internal mammary (IM) adenopathy.

She received four cycles of dose dense doxorubicin/cyclophosphamide (ddAC) at 70/700 mg/m^2^, later reduced to 60/600 mg/m^2^ for an additional three cycles due to urinary retention, followed by a left palliative mastectomy requiring wound vacuum-assisted closure intraoperatively. Surgical pathology revealed an 8 cm CC by 20 cm TRANS by 8 cm AP grade three IDC with positive lateral and deep surgical margins. The tumor cells were characterized by high nuclear-to-cytoplasmic ratio, prominent nucleoli, irregular nuclear contours, hyperchromasia, and frequent mitoses, with extensive dermal lymphatic invasion.

**Figure 1:**
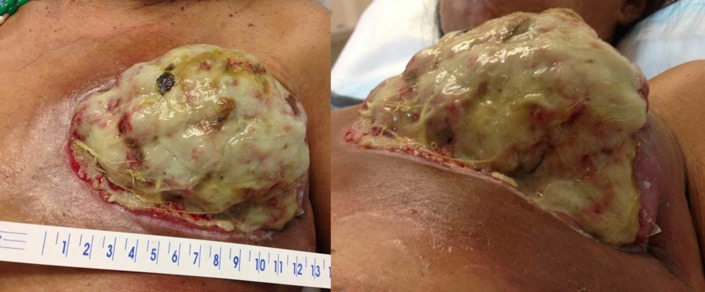
Appearance of the recurrent left chest wall mass at the time of presentation to our institution eight weeks after completion of palliative left mastectomy with positive surgical margins.

Following mastectomy, she had rapid regrowth of the chest wall tumor over the following eight weeks and at that time presented to our institution for management. On physical exam, she had a massive, fungating left chest wall mass arising from the mastectomy scar measuring greater than 12 cm in greatest dimension (Figure 1) with extensive erythema, drainage, and foul odor with associated tachycardia and fever suggestive of superinfection. She was admitted for management and started on broad spectrum antibiotics with resolution of infectious symptoms.

**Figure 2:**
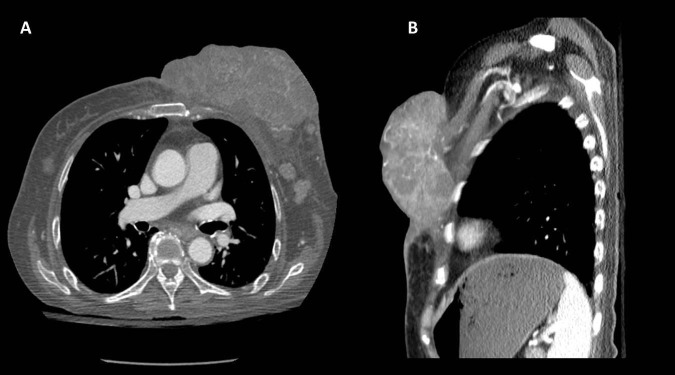
(A) Cross-sectional and (B) sagittal contrast-enhanced computed tomography (CT) imaging demonstrating the large left chest wall mass inseparable from the left pectoralis major muscle. Large mediastinal, axillary, and chest wall adenopathy can also be seen.

CT chest imaging at that time showed a 13 cm lobulated left breast mass with heterogeneous contrast enhancement inseparable from the thin left pectoralis muscle and poorly defined mediastinal, left axillary, and chest wall lymphadenopathy (Figures 2A-2B). CT scan of the abdomen and the pelvis, and magnetic resonance imaging (MRI) of the brain showed no evidence of active metastatic disease. Tumor and germline genomic testing from the initial biopsy revealed the following alterations: STK11 D194N, NMYC amplification, IRS2 amplification, TP53 F113fs*5, NOTCH1 V1575del – subclonal, RB1 loss, KDM5A amplification – equivocal, and FGF14 amplification.

**Figure 3:**
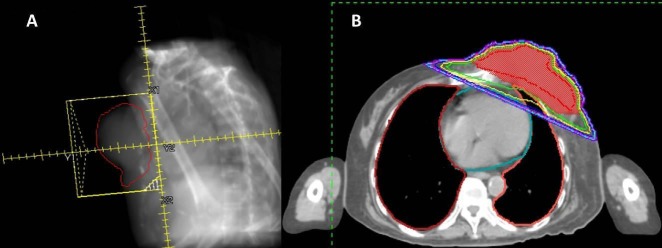
(A) Beam’s eye view (BEV) digitally reconstructed radiograph (DRR) of opposed tangential 6 megavoltage (MV) photon radiation fields. (B) Representative cross-sectional computed tomography (CT) planning image showing radiation isodose coverage of the tumor.

After multidisciplinary discussion, she was offered palliative concurrent hypofractionated chemoradiation (CRT) with carboplatin area under the curve (AUC) dosing of five and paclitaxel dosing of 175 mg/m^2^ every three weeks for six cycles with 45 Gy left chest wall radiation delivered over 15 daily fractions (Monday through Friday) of 3 Gy each using opposed tangential 6 megavoltage (MV) photon radiation fields prescribed to an effective point. The beam’s eye view (BEV) digitally reconstructed radiograph (DRR) is shown in Figure 3A, and a representative cross-sectional image showing radiation isodose tumor coverage is shown in Figure 3B.

**Figure 4:**
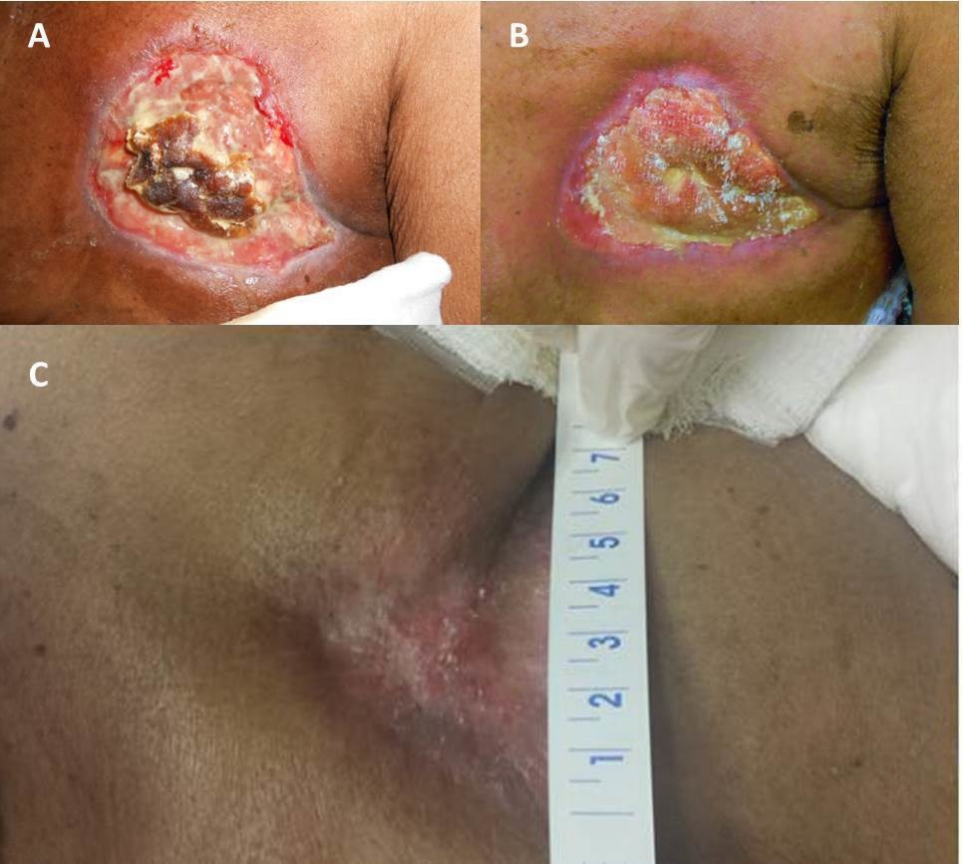
Appearance of the left chest wall (A) three weeks, (B) nine weeks, and (C) 13 months following the completion of concurrent chemoradiation (CRT).

**Figure 5:**
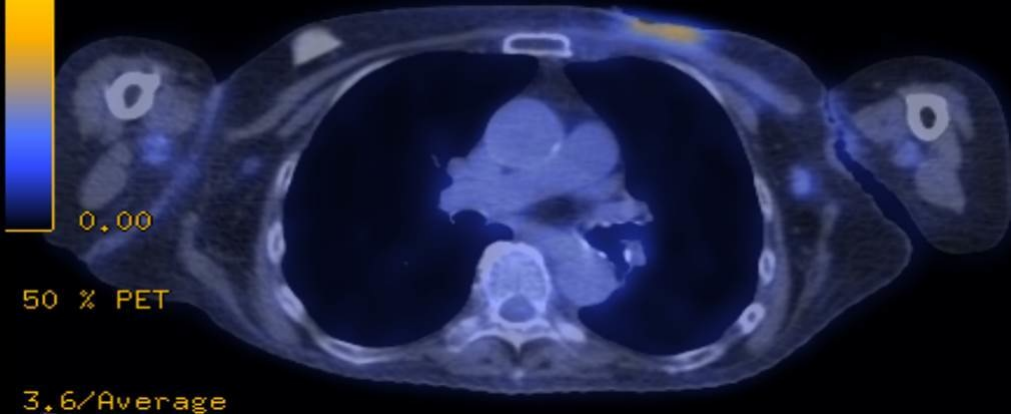
Cross-sectional positron emission tomography/computed tomography (PET/CT) imaging at 18 months post chest wall radiation showing ongoing local control of the chest wall disease.

During CRT, the patient developed the expected grade two dermatitis of the surrounding skin and modest hematologic toxicities. She had no grade three or greater toxicities during CRT and had significant shrinkage of the fungating mass by completion of 45 Gy, with continued shrinkage over the next three weeks. On clinical exam three weeks following the completion of CRT, the large fungating mass had been replaced by a wound with scant bleeding, extensive debris and granulation tissue (Figure 4A). By nine weeks post-treatment, the wound was no longer bleeding and new skin growth was noted around the periphery (Figure 4B). She continued wound care and had continued granulation and healing of the chest wall wound. By four months post-treatment, she had only mild chest wall pain responsive to acetaminophen. Fine needle aspiration of the wound performed seven months following completion of radiotherapy (RT) showed no evidence of malignant cells. Figure 4C shows the wound at 13 months following completion of RT, and Figure 5 shows a representative cross-sectional image from a positron emission tomography/computed tomography scan (PET/CT) taken 18 months following chest wall RT, demonstrating continued local control.

In hopes of enrolling on clinical trial, the patient had repeat tumor testing done by a certified lab that revealed the tumor to be, in contrast to the initial pathology, ER positive, and the patient initiated endocrine therapy with letrozole. Her disease remained well-controlled for 10 months on endocrine therapy when surveillance PET/CT showed low-volume systemic disease progression with small pulmonary and liver nodules and increase in size and fluorodeoxyglucose (FDG) avidity of left axillary lymph nodes without associated chest wall progression. She subsequently underwent treatment with additional systemic therapy on clinical trial using an antibody-drug conjugate, before ultimately succumbing to complications of brain metastases at 22 months following completion of CRT. At the time of her death, her chest wall disease remained well-controlled, although she required an additional course of palliative RT for a growing axillary node outside the initial RT field 17 months after her initial RT course.

## Discussion

Worldwide, breast cancer is the leading female cancer diagnosis and the leading cause of cancer death in women [7]. Inflammatory breast cancer is a rare, aggressive, poorly understood subset of invasive breast cancers comprising less than 1% of all breast cancer diagnoses estimated to be nearly 250,000 cases in the US in 2016 [1]. The disease is a “clinical-pathologic” diagnosis typified by less than six months of diffuse breast erythema, swelling, and edema (*peau d’orange*). Unfortunately, given the rarity of the disease and aggressive biologic behavior, many patients present with incurable metastatic disease. Current trials seek to identify actionable molecular targets, combine systemic and immunomodulatory drugs, or are investigational, and long term overall survival remains decidedly poor.

In the case presented, a hypofractionated radiotherapy regimen was employed with concurrent full-dose carboplatin/paclitaxel to control a massive, rapidly growing chest wall recurrence. Treatment approaches for unresectable chest wall recurrence are not standardized, and limited data addresses the use of concurrent chemoradiation for these patients. Zagar, et al. describe results for 27 patients treated with concurrent single agent chemotherapy with conventionally fractionated (1.8–2.0 Gy/fraction) radiation and hyperthermia for unresectable chest wall recurrence, with one-year local progression-free survival of 76% [8]. Karawasa, et al. enrolled 35 patients with recurrent or unresectable breast cancer in a small prospective study using concurrent radiation (2–3 Gy/fraction; if 3 Gy fractions were used, treatment was three times weekly) with single agent docetaxel, with an encouraging complete response rate of 68% [9].

Our patient’s treatment regimen was novel in both the use of daily hypofractionated radiation and concurrent multi-agent chemotherapy. Hypofractionation in this setting is radiobiologically appealing. Breast cancer is thought to have a low alpha/beta (α/β) ratio meaning that tumor cells have the capacity to repair sublethal DNA damage, thus longer cell cycle times, fewer mitoses, less radiosensitivity, and late responding tissue toxicity [10]. By increasing the fractional radiation dose, logarithmic cell kill becomes less dependent upon cell death due to sublethal damage (β), thus improving radiosensitivity. To this end, hypofractionated adjuvant whole breast radiation has been explored in a number of large randomized clinical trials demonstrating excellent clinical outcomes [10]. Second, this patient was treated with concurrent full dose carboplatin and paclitaxel, both of which act to modulate the radioresponsiveness of tumor cells, thus increasing the α/β ratio and further improving logarithmic cell kill. Carboplatin/paclitaxel is a widely used partner regimen to RT in other disease settings such as lung cancer and is typically used in attenuated, radiosensitizing doses with paclitaxel at 45–50 mg/m^2^ and carboplatin at an AUC of two. Our patient received concurrent, full dose chemotherapy with paclitaxel at 175 mg/m^2^ and carboplatin at an AUC of five. Concurrent full dose chemotherapy poses a potential risk of synergistic lung toxicity; however, our patient had limited lung within the RT fields and did not develop any symptoms suggestive of pneumonitis. Due to the patient’s anatomy, her lung doses were low, with the ipsilateral volume of lung receiving 20 Gy (V20) of 4.8% and a mean cardiac dose of 3.4 Gy. Our regimen may prove more toxic in patients for whom adequate lung sparing cannot be achieved.

Finally, it is worth commenting on the observed histopathologic and genomic aberrations in this unique case. Clinically, the patient’s tumor was characterized by aggressive growth kinetics with a 12 cm chest wall recurrence only eight weeks after mastectomy. This is consistent with the surgical histopathology, which revealed a high-grade tumor typified by bizarre appearing nuclei, with frequent mitoses and rapid cellular proliferation (Ki-67 of 33%), and aggressive features (dermal lymphatic invasion). In regards to the genomic alterations, the striking feature is not necessarily the specific observed aberrations, but rather, the combined number and synergism of known molecular drivers of disease leading to the aggressive tumor phenotype. STK11 is a tumor suppressor serine/threonine-protein kinase that is involved in regulating apoptosis, DNA damage response, and inhibition of signaling pathways that promote cell growth and proliferation. NMYC is a proto-oncogene with broad genomic transcriptional activity, and amplification leads to abnormalities in regulation of cellular proliferation and apoptosis. IRS2 is an insulin receptor signaling molecule that has implications on cellular metabolism and broad receptor tyrosine kinase effects. TP53 and RB1 are tumor suppressor genes that are broadly implicated in human malignancies and regulate cell growth by inhibiting cell cycle progression. NOTCH1 is a transmembrane receptor and is involved in cell survival signaling. FGF14 is a fibroblast growth factor and is involved in cell survival and replication. Taken together, the patient's tumor had dysregulating alterations in multiple tumor suppressor and proto-oncogenes, which likely led to the highly aggressive clinical growth. Unfortunately, despite the ability to detect and characterize these genomic alterations, there are currently no approved systemic therapies to target these drivers of disease, and ongoing pharmaceutical development of targeted agents is necessary.

## Conclusions

Unresectable chest wall recurrence from inflammatory breast cancer represents a unique treatment challenge with significant morbidity and mortality. The limited available data suggests that concurrent chemoradiation is a promising approach for achieving durable local control of disease, and our patient’s remarkable response to hypofractionated concurrent chemoradiation with full dose carboplatin and paclitaxel suggests this approach warrants additional investigation.
